# Causal associations between gut microbiota and urological tumors: a two-sample mendelian randomization study

**DOI:** 10.1186/s12885-023-11383-3

**Published:** 2023-09-11

**Authors:** Wang Mingdong, Gao Xiang, Quan Yongjun, Wang Mingshuai, Ping Hao

**Affiliations:** 1 Department of Urology, Beijing Tongren Hospital, Capital Medical University, Beijing, China; 2 Department of Otolaryngology Head and Neck Surgery, Beijing Tongren Hospital, Capital Medical University, Beijing, China; 3 Department of Urology, Cancer Hospital, Chinese Academy of Medical Sciences, Beijing, China

**Keywords:** Gut microbiota, Bladder cancer, Prostate cancer, Kidney cancer, Mendelian randomization, FinnGen

## Abstract

**Background:**

Dysbiosis of gut microbiota has been linked to numerous diseases, including cancer. The unique role of gut microbiota in urological tumors is gaining prominence. However, it is still controversial whether the dysbiosis of gut microbiota should be one of the etiological factors of bladder cancer (BCa), prostate cancer (PCa) or kidney cancer (KCa).

**Materials and methods:**

The microbiome genome-wide association study (GWAS) from the MiBioGen consortium (18,340 samples of 24 population-based cohorts) was utilized as the exposure data. Additionally, outcomes data (951 BCa cases and 307,092 controls; 1,631 KCa cases and 238,678 controls; 79,148 PCa cases and 61,106 controls) were extracted from the GWAS of the FinnGen and PRACTICAL consortia. To detect the potential causative bacterial traits for BCa, PCa, and KCa, a two-sample Mendelian randomization (MR) analysis was performed, employing the inverse-variance weighted or Wald ratio method. Sensitivity analyses were subsequently conducted to explore the robustness of the primary results. Finally, the reverse MR analysis was undertaken to mitigate the reverse causation.

**Results:**

This study suggested that *Bifidobacterium* (*p* = 0.030), *Actinobacteria* (*p* = 0.037 for phylum, 0.041 for class), and *Ruminococcustorques group* (*p* = 0.018), exhibited an association with an increased risk of BCa using either the inverse-variance weighted or Wald ratio method. By utilizing the Wald ratio method, *Allisonella* (*p* = 0.004, *p* = 0.038) was associated with a decreased risk of BCa and PCa, respectively. Furthermore, *Ruminococcustorques group* (*p* = 0.028) and *Erysipelatoclostridium* (*p* = 0.048) were causally linked to an elevated risk of KCa.

**Conclusions:**

This MR study supports that genetically predicted gut microbiota is causally related to BCa, PCa and KCa. Additionally, distinct bacterial traits are identified in relation to each tumor type.

**Supplementary Information:**

The online version contains supplementary material available at 10.1186/s12885-023-11383-3.

## Introduction

Urological tumors pose a severe threat to human life and health on a global scale. According to the Global Burden of Disease Study (2019), the incidence rates of bladder cancer (BCa), prostate cancer (PCa), and kidney cancer (KCa) stand at 6.5, 17.4, and 4.6 per 100,000 person-years, collectively accounting for 9.81% of the worldwide tumor incidence. The mortality rates of PCa, BCa, and KCa are respectively 6.3, 2.9, and 2.1 per 100,000 person-years, contributing to approximately 9.14% of all tumor-related fatalities [1]. Furthermore, variations in incidence rates are observed globally, with higher incidence rates in industrialized nations like the United States and Europe. Meanwhile, the incidence rates of South American countries are higher than rising nations like African and Asian [2–4]. In recent decades, with the application of immunotherapy in tumors, focusing on the organism immunity of tumors and the various factors that regulate the immune status is increasing, and gut microbiota is one of them [5]. Since the Human Microbiome Project launched, there has been increasing evidence to suggest that the microbiome, including urinary tract, gut, and intratumoral microbes, contributes to urologic tumorigenesis [6].

Recently, gut microbiota has gained widespread attention as a remarkable factor to regulate the health of organism. Dysbiosis of gut microbiota can regulate immune, energy, lipid and glucose metabolism pathways that involved in the development of diseases, such as obesity, type 2 diabetes, hepatic steatosis, and cancer [7]. The gut microbiota plays an important role in the development of various gastrointestinal disorders, including colorectal cancer and inflammatory bowel disease, for which digestive tract is habitat of gut microbiota [8]. With organs indirect contact with each other, it remains unclear whether a relevancy exists between gut microbiota and urinary system. According to some preliminary investigations, gut microbiota affects the growth of PCa, BCa, and KCa through their metabolites [9–11]. Matsushita M et al. reported that short-chain fatty acids (SCFAs) producing bacteria, namely *Rikenellaceae*, *Alistipes*, and *Lachnospira*, of those which were considerably increased in men with high Gleason prostate cancer [12]. He et al. demonstrated that among BCa patients, *Prevotella, Clostridium cluster XI*, and the concentration of butyric acid in feces were significantly reduced [13]. Dai et al. reported that the disturbance of tryptophan metabolites in gut microbiota is associated with renal cancer metastasis [11]. However, due to the scarcity of large-scale real-world studies, these observations remain to be definitively substantiated. Furthermore, investigations into gut microbiota are inherently challenging and resource-intensive, requiring advanced molecular tools and techniques (i.e., macrogenome, metabolome, lipidome, and macrotranscriptome) while grappling with the complexities of confounding factors, biases, and reverse causation in general observational studies.

Genome-wide association studies (GWAS) detect genetic variants across large populations to verify phenotype-genotype associations, and more than 50,000 associations of genome-wide significance have been reported in various common diseases and traits [14]. MR is a statistical method that leverages GWAS data as distinct phenotypes to address the limitations of observational studies. By considering genetic variants as instrumental variables (IVs), MR seeks to uncover the potential causal links between exposures and outcomes [15]. MR has been widely applied in research on causal inference. Recently, MR investigations have revealed the causality between gut microbiota and several illnesses such as colorectal cancer, Alzheimer’s disease, autoimmune diseases, or psychiatric disorders [16–19].

This study aims to investigate the potential causality between gut microbiota and urological tumors, specifically prostate cancer, bladder cancer, and renal cancer, by employing the two-sample MR method.

## Materials and methods

### Study design overview

This study employs the two-sample MR method to investigate the causal associations between gut microbiota and PCa, BCa, and/or KCa. Summary statistic data for gut microbiota and PCa, BCa, or KCa were extracted from the substantial GWASs to select the IVs. Under the condition of published research and open-access summary data were used, thus further ethical approval or participant consent was unnecessary.

**Fig. 1:**
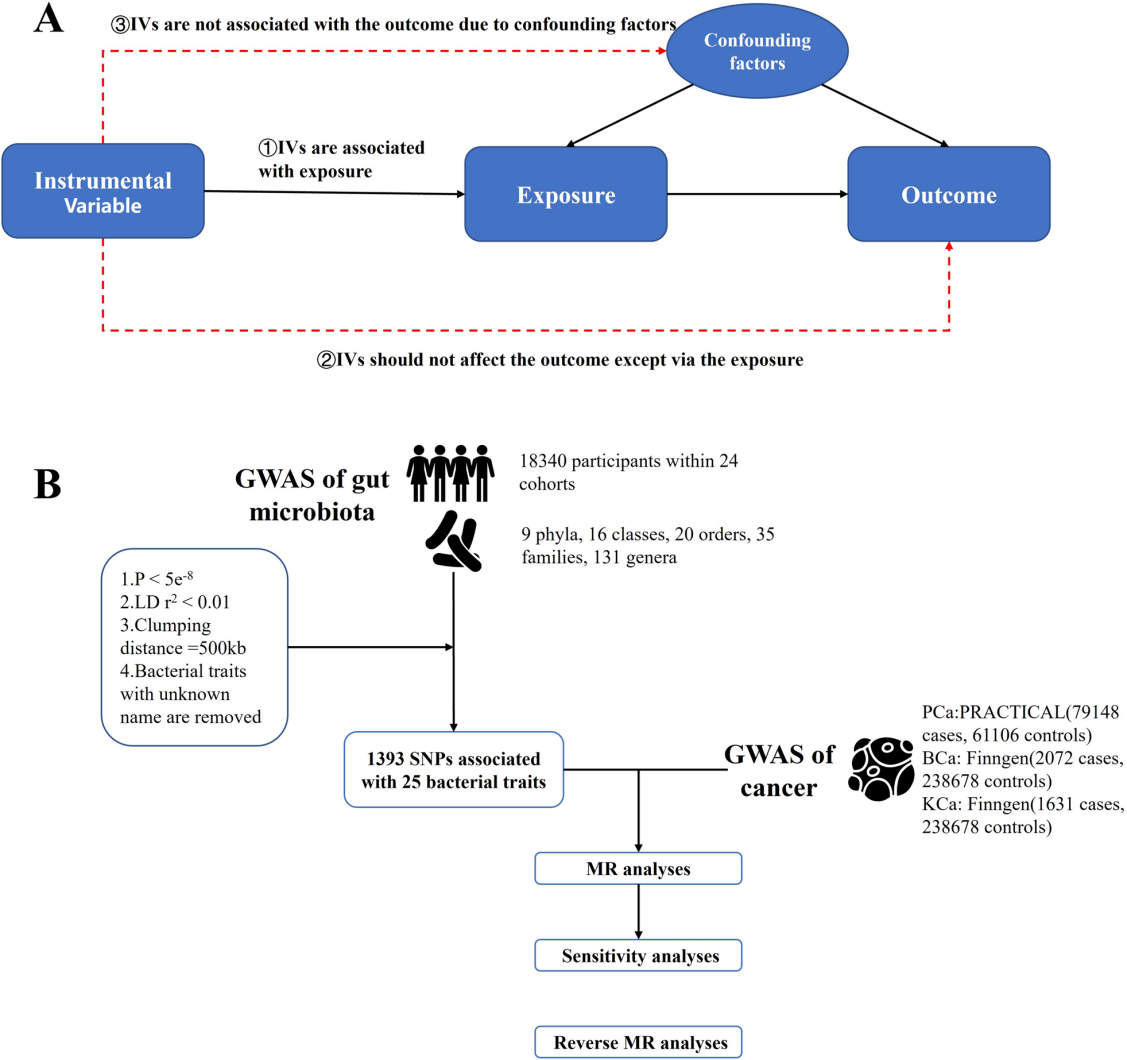
The overview of design. IVs: instrumental variables; GWAS: Genome Wide Association Study; LD: linkage disequilibrium; SNP: single nucleotide polymorphism; BCa: bladder cancer; PCa: prostate cancer; KCa: kidney cancer

To mitigate the potential biases on the results, adherence to three major assumptions in MR method is crucial: (1) IVs must exhibit a significant association with the exposure [20]; (2) IVs should exclusively influence the outcome via the exposure [20]; and (3) IVs must not be linked to the outcome due to confounding factors [20]. An overview of the study design is illustrated in Fig. 1.

### Date sources

#### Gut microbiota

A large-scale GWAS involved 18,340 participants from 24 cohorts provides summary statistics for gut microbiota, using 16 S rRNA gene sequencing [21]. Available data were from the MiBioGen (https://mibiogen.gcc.rug.nl/). In all, 211 traits (131 genera, 35 families, 20 orders, 16 classes, and 9 phyla) were included [21]. First, IVs were selected at *p* < 5 × 10^−8^ to meet the stricter threshold. Subsequently, linkage disequilibrium (LD) clumping was executed to mitigate LD among single nucleotide polymorphisms (SNPs) (r^2^ < 0.001, distance = 10,000 kb), resulting in an adjusted cutoff (r^2^ < 0.01, distance = 500 kb) to retain a viable number of SNPs for analysis. SNPs without attribution to specific bacterial traits were excluded. Finally, “PhenoScanner” (http://www.phenoscanner.medschl.cam.ac.uk/) was used to exclude SNPs that were clearly associated with risk factors of the urologic tumors. A total of 1,393 SNPs closely related to 25 bacterial traits were incorporated into the MR analysis.

#### PCa, BCa, and KCa

Summary statistics for PCa (79,148 cases and 61,106 controls of European descent) were furnished by the Prostate Cancer Association Group to Investigate Cancer-Associated Alterations in the Genome (PRACTICAL, http://practical.icr.ac.uk) consortium [22]. FinnGen research project (https://www.finngen.fi), which involves participates of European descent, provided summary statistics for BCa (2,072 cases, 238,678 controls,) and KCa (1,631 cases, 238,678 controls) [23].

### Statistical analysis

F-statistics were employed to test the strength of IVs, mitigating potential weak instrument bias which could confound causal association estimates. F-statistics were calculated through the following formula: F = R^2^ (n-k-1)/k(1-R^2^), where “n” signifies the sample size, “k” denotes the number of IVs and “R^2^” represents the portion of exposure variance elucidated by the IVs [24]. Generally, R^2^ was estimated via employing the equation required minor allele frequency (MAF) and β value: R^2^ = 2 × MAF × (1 − MAF) × β^2^. Due to the lack of MAF value in GWAS, the function “get_r_from_bsen” of “TwoSampleMR” package was employed for R^2^ estimation. The weak IVs were discarded with F-statistics < 10.

In this study, the MR analysis involving bacterial traits linked to individual SNPs was conducted using the Wald ratio method. Multiple tests, encompassing the inverse variance weighted (IVW) method, weighted median method, and MR-Egger regression test, were conducted for bacterial traits with multiple associated SNPs [25]. The results of the IVW method were plausible if the three assumptions of MR were satisfied for SNPs [25]. The Cochrane’s Q test was performed to scrutinize SNP-associated heterogeneity for each bacterial trait. In cases where significant heterogeneity emerged (*p* < 0.05), a fixed-effects IVW model was applied; contrarily, a random-effects IVW model was applied [26]. Additionally, MR-Egger intercept test and leave-one-out analysis were performed for sensitivity analysis [27]. The *p*-value of MR-Egger intercept test functioned as an indicator of the horizontal pleiotropy (statistically significant if *p* < 0.05) [28]. Leave-one-out analysis was used to discern potential pleiotropic effects stemming from individual SNP. Finally, reverse MR analysis was executed to verify the existence of reverse causality between BCa, PCa, KCa, and gut microbiota. All MR analyses were conducted utilizing “TwoSampleMR” R package (version 4.2.1).

## Results

### Overview of instrumental variables

Following a sequence of rigorous quality control procedures, 27 SNP (*p* < 5 × 10^−8^, R^2^ < 0.01) associated with 19 bacterial traits were selected as IVs for analysis (Supplementary Table 1). The IVs employed in the MR analysis possessed F-statistics within a range of 30.07 to 200.70, all of which exceeded the threshold of > 10. This indicates a robust instrument strength and mitigates the potential impact of weak instrument bias (Supplementary Table 2). Limited by the number of available IVs, the sensitivity analysis was restricted to the *Bifidobacterium*. Notably, the statistical effect size remained relatively consistent across taxonomic levels, encompassing order, family, and genus.

### Gut microbiota and BCa

**Fig. 2:**
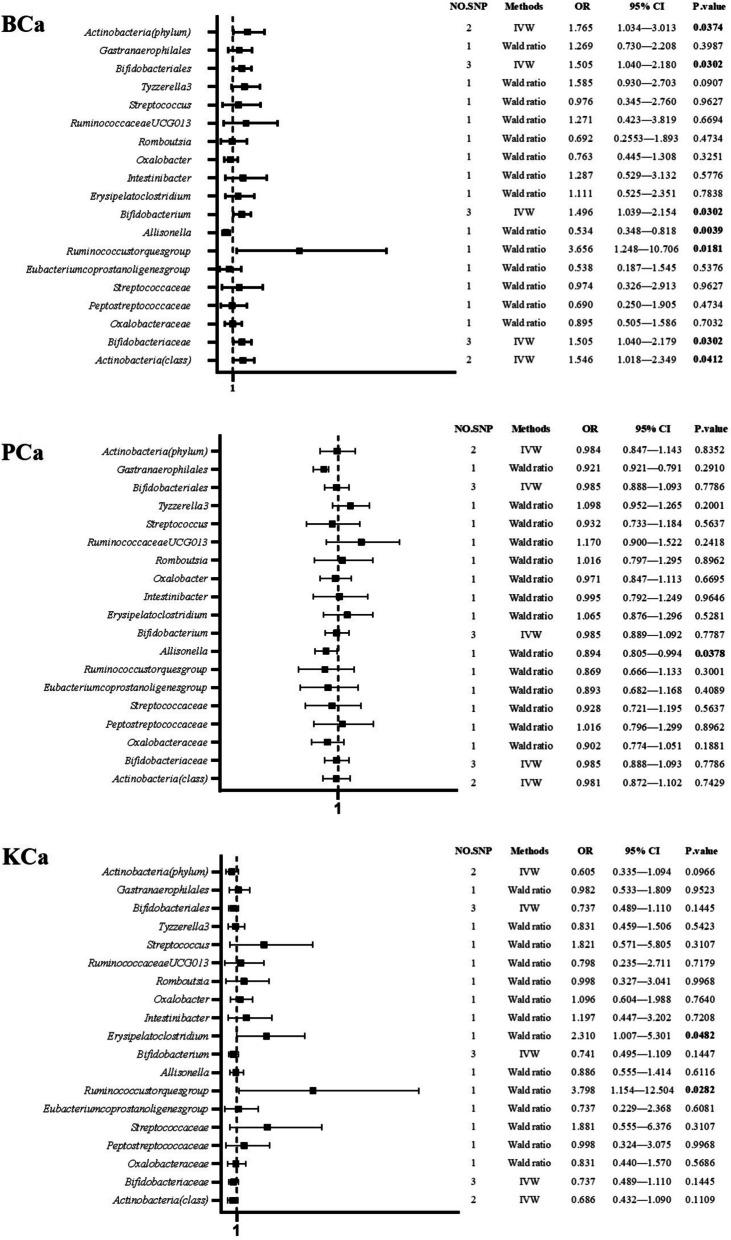
The forest plots of major results. SNP: single nucleotide polymorphism; BCa: bladder cancer; PCa: prostate cancer; KCa: kidney cancer; IVW: inverse-variance weighted; OR: odds ratio

In the context of MR analysis, a comprehensive assessment revealed that 7 bacterial traits (encompassing various taxonomic levels of phylum, class, order, family, and genus) exhibited statistically associations with the risk of BCa. This suggests a potential role for specific bacterial traits in the etiology of BCa (Table 1; Fig. 2).

**Table 1:** Significant MR analysis results

Outcomes	Bacterial traits (rank)	N.SNP	F	Methods	OR	95% CI	P.val
BCa	Actinobacteria (class)	2	166.914	IVW	1.546	1.018—2.349	0.041
	Bifidobacteriaceae (family)	3	196.473	IVW	1.505	1.040—2.179	0.030
				Weighted median	1.522	1.005—2.305	0.047
	Ruminococcustorquesgroup	1	31.285	Wald ratio	3.656	1.248—10.706	0.018
	Allisonella	1	32.374	Wald ratio	0.534	0.348—0.818	0.004
	Bifidobacterium (genus)	3	200.670	IVW	1.496	1.039—2.154	0.030
				Weighted median	1.512	1.011—2.260	0.044
	Bifidobacteriales (order)	3	196.473	IVW	1.505	1.040—2.180	0.030
				Weighted median	1.522	1.012—2.290	0.044
	Actinobacteria (phylum)	2	112.438	IVW	1.765	1.034—3.013	0.037
PCa	Allisonella (genus)	1	32.374	Wald ratio	0.894	0.805—0.994	0.038
KCa	Ruminococcustorquesgroup (genus)	1	31.285	Wald ratio	3.798	1.154—12.504	0.028
	Erysipelatoclostridium (genus)	1	34.619	Wald ratio	2.310	1.007—5.301	0.048

*BCa *Bladder cancer, *PCa *Prostate cancer, *KCa *Kidney cancer, N. SNP, the number of SNPs used as IVs. *SNP *Single-nucleotide polymorphism, *OR *Odds ratio, *CI *Confidence interval, *MR *Mendelian randomization, *IVW *Inverse-variance weighted; Significant P was marked in bold

Among the aforementioned traits, *Bifidobacterium*, *Bifidobacteriaceae*, *Bifidobacteriales* were found to be the same category of bacteria, sharing identical IVs represented by rs182549, rs7322849, and rs7570971. As demonstrated in Table 1, the IVW analysis was concluded that *Bifidobacterium* exhibited a causal relationship with an elevated risk of BCa (OR: 1.496, 95% CI: 1.039–2.154, *p* = 0.030); similar results were obtained for *Bifidobacterium* and *Bifidobacteriaceae* (OR: 1.505, 95% CI: 1.040–2.179, *p* = 0.030; OR: 1.505, 95% CI: 1.040–2.180, *P* = 0.030, respectively). The results also remained robust in weighted median analyses (OR: 1.512, 95% CI: 1.011–2.260, *p* = 0.044; OR: 1.522, 95% CI: 1.005–2.305, *p* = 0.047; OR: 1.522, 95% CI: 1.012–2.290, *p* = 0.043, respectively). The causal relationship observed within the higher taxonomic level of *Bifidobacteria*, specifically *Actinobacteria* (phylum) and *Actinobacteria* (class), echoed the findings with an increased risk of BCa (OR: 1.765, 95% CI: 1.034–3.013, *p* = 0.037; OR: 1.546: 95% CI: 1.018–2.349, *p* = 0.041, respectively). Additionally, *Ruminococcustorques group* and *Allisonella*, each had only one SNP, exhibited potential causal relationships as determined by Wald ratio analyses (OR: 3.656, 95% CI: 1.248–10.706, *p* = 0.018; OR: 0.534, 95% CI: 0.348–0.818, *p* = 0.004, respectively).

In the subsequent analyses, Cochran’s Q test did not reveal any statistically significant heterogeneity for any of the applicable bacterial traits (*p* > 0.10, Supplementary Table 3). A leave-one-out analysis was applied and identified no single SNP with a significant influence on the IVW estimate (Supplementary Fig. 1). Furthermore, less directional pleiotropy was inspected in the MR-Egger test (*Bifidobacterium*, intercept *p* = 0.964; *Bifidobacteriaceae*, intercept *p* = 0.944; *Bifidobacteriales*, intercept *p* = 0.944, Supplementary Table 4). Considering the absence of enough SNPs, a sensitivity analysis is not feasible for other bacterial traits.

### Gut microbiota and PCa or KCa

Genetical evidence indicated a negative effect of *Allisonella* on the risk of PCa (OR: 0.894, 95%CI: 0.805–0.994, *p* = 0.038) as determined through Wald ratio analysis (Table 1; Fig. 2). In parallel, *Ruminococcustorques group* and *Erysipelatoclostridium* were causally associated with an elevated risk of KCa (OR: 3.798, 95% CI: 1.154–12.504, *p* = 0.028; OR: 2.310, 95% CI: 1.007–5.301, *p* = 0.048, respectively) as indicated by Wald ratio analysis (Table 1; Fig. 2). There were not enough SNPs to conduct a sensitivity analysis for the aforementioned bacterial traits.

### Reverse MR analysis

Adopting a consistent threshold with the main analysis for SNP selection, reverse MR analysis failed to identify any causal relationships between BCa, PCa, KCa, and gut microbiota (Supplementary Tables 5, 6).

## Discussion

This study pioneers the utilization of a two-sample MR approach to identify a potential causal relationship between specific gut microbiota taxa and three major urological tumors: BCa, PCa, and KCa, which provides directions for further mechanistic investigations.

The human microbiota resides ubiquitously across the body surface and natural cavities, forming a harmonious symbiotic equilibrium [1]. Therefore, the intricate interplay between human microbiota and organismal health has long been a focal point of research. Numerous existing researches have revealed the potential influence of urinary system bacteria on urological tumor development, the widespread concern in the unique role of gut microbiota in neoplastic diseases has sparked novel inquiries into the relationship between gut microbiota and urological tumors [29, 30].

This two-sample MR analysis unveiled a surprising increase in the risk of BCa associated with *Bifidobacterium* at the order, family, and genus levels, *Actinobacteria* at the phylum and class levels, *Ruminococcustorques group* at the genus level, while *Allisonella* at the genus level appeared to confer a potential protective effect against BCa. *Bifidobacterium* has long been recognized as a probiotic abundant in fermented dairy products. Studies have indicated that the intake of fermented dairy foods is linked to a reduced risk of BCa [31]. *Bifidobacterium* may play a role in the regulation of proliferation, apoptosis, responses to immune therapy, radiation, and chemotherapy [32]. However, existing research on the anticancer benefits of probiotics are primarily concentrated on the intestinal tract tumors, with a lot of unknown of their impact on tumors in other organs [32]. Given that the interactions in the intestinal tract are more direct and intricate due to the site’s flora aggregation, it remains questionable whether the preliminary findings regarding *Bifidobacterium*, or even probiotics in general, and their effects on intestinal tumors can be extrapolated to tumors in other organs. Moreover, *Lactobacillus*, including *Bifidobacterium*, has been implicated in promoting the pathogenesis of gastric cancer through diverse mechanisms, including supplying exogenous lactic acid, stimulating inflammation, angiogenesis, and epithelial-mesenchymal transition [33]. Thus, this study has a proposal in a novel avenue of etiological evidence indicating that *Bifidobacterium* could potentially contribute to BCa development. At present, a dearth of research exists regarding the specific mechanism underlying the relationship between *Bifidobacterium* and BCa. Future exploration is essential to determine whether intestinal *Bifidobacterium* influences critical physiological activities of urothelial cells through various small molecular metabolites or via translocation and colonization of the urinary tract. Speculatively, based on current knowledge, *Bifidobacterium* may possess the ability to stimulate macrophages, T lymphocytes, and epithelial cells to secrete tumor necrosis factor α, which in turn could promote tumor proliferation, survival, and evasion from immune surveillance—this represents a potentially promising mechanistic pathway [34, 35]. In terms of observational studies, a preliminary study of gut microbiota in BCa patients, comprised 26 cases and 16 health controls, reported decreased gut microbial diversity at the phylum level, with decreased relative quantities of *Clostridium cluster XI* and *Prevotella* in BCa patients; however, no significant difference was observed in relation to *Bifidobacterium *[13]. Emerging evidence suggests that gut microbiota could influence BCa treatment outcomes. Non-muscle invasive BCa patients treated with probiotics exhibited lower recurrence rates [36]. Recently, the first fecal macro-genomic research of BCa was published, encompassing 32 cases and 15 health controls, confirmed the viewpoint of gut microbiota dysbiosis in BCa and revealed changes in key metabolites. Furthermore, the relative abundance of 19 microbiota at the genus level (including *Bifidobacterium*) was diminished in fecal samples from BCa patients [37]. Besides the unclear reverse causality and limited sample size, the inclusion and exclusion criteria which only excluded vegetarians, failed to account for the potential confounding introduced by various dietary habits, and the data of vegetarians were omitted. Consequently, the identification of relevant discrepancies necessitates further investigation.

This study also identified that at the genus level, *Allisonella* decreased the risk of PCa. The intricate interplay between gut microbiota and PCa has recently garnered significant attention, leading to the emergence of “gut-prostate axis” concept. However, the precise mechanisms remain elusive. Serveral observational studies have individually suggested potential associations between PCa and various bacteria, including *Streptococcus, Bacteroides, massiliensis, Prevotella 9, Erysipelotrichaceae, Escherichia/Shigella, Rikenellaceae, Alistipes or Lachnospira *[12, 38–40]. This study uniquely highlighted the role of *Allisonella* while meticulously addressing reverse causality and confounding issues. Metabolites produced by gut microbiota, such as SCFAs, may regulate PCa growth, and more significantly, androgen production by gut microbiota could contribute to the development of castration-resistant prostate cancer [9]. Due to the intricate nature of gut microbiota, causality should be interpreted cautiously; nonetheless, the undeniable significance of gut microbiota in the evolution of prostate cancer is evident.

*Ruminococcustorques group* and *Erysipelatoclostridium* at genus level were identified as potential contributors to KCa occurrence. The sole available observational study (comprising 50 cases and 40 health controls) reported positive associations between *Blautia, Streptococcus, Ruminococcustorques_group, Romboutsia*, and *Eubacteriumhallii group* with renal clear cell cancer. This study also indicated that *Streptococcus* promotes renal clear cell cancer progression in vitro through the TGF-β signaling pathway [41]. While consensus exists regarding the potential involvement of the *Ruminococcustorques* group in the occurrence of KCa, the substantiation of this correlation necessitates more robust empirical evidence.

Several limitations are existed in this study. Firstly, the findings are limited to European lineages, thus the substantial variations in gut microbiota composition across different populations were not considered. Secondly, the 16 S rRNA gene sequencing method only allowed discrimination from the phylum level to the genus level and not at a more specific taxonomic level. Thirdly, it is unfit to conduct stratified MR due to the unavailability of population basic characteristics, such as gender, culture, occupation, etc., which bring about a lack of explanatory power for the hazards associated with particular populations. Finally, gut microbiota may be influenced by dietary habits or other environmental factors, yet they were unable to assess whether genetic instrumental variables were correlated with these confounding factors due to the unavailability of relevant information.

## Conclusions

This MR study supports genetic evidence that gut microbiota is causally related to BCa, PCa or KCa, and specific bacterial traits are suggested separately. The results offer a fresh perspective for exploring the intricate mechanisms through which gut microbiota affects urological tumors.

### Supplementary Information

**Additional file 1: Supplementary Table 1.** Detailed information of instrumental variables used in MR analyses. **Supplementary Table 2.** MR analysis of all gut microbiome. **Supplementary Table 3.** The heterogeneity results from the Cochran's Q test. **Supplementary Table 4.** Directional pleiotropy results from Egger intercept analysis. **Supplementary Table 5.** Detailed information of instrumental variables used in reverse MR analyses. **Supplementary Table 6.** Reverse MR analysis of all gut microbiome

**Additional file 2: Supplementary Figure 1.** Leave-one-out analysis results of *Bifidobacteriales(order)*,*Bifidobacteriaceae(family)*, and,* Bifidobacterium(genus)*

## Data Availability

The original statistical data analyzed in the study are presented in the article, which is open access.

## Abbreviations

MR: Mendelian randomization
BCA: Bladder cancer
PCA: Prostate cancer
KCA: Kidney cancer
GWAS: Genome-wide association
IVs: Instrumental variables
LD: Linkage disequilibrium
MAF: Minor allele frequency
SNP: Single nucleotide polymorphism
IVW: Inverse variance weighted
SCFAs: Short-chain fatty acids
