# Supplementary material for: Causal associations between gut microbiota and urological tumors: a two-sample mendelian randomization study
Source: BMC Cancer. 2023 Sep 11;23:854. doi: 10.1186/s12885-023-11383-3 (PMC10496293; doi:10.1186/s12885-023-11383-3)
Supplement: Supplementary file 2 — Additional file 2: Supplementary Figure 1. Leave-one-out analysis results of Bifidobacteriales(order),Bifidobacteriaceae(family), and, Bifidobacterium(genus) [file 12885_2023_11383_MOESM2_ESM.docx]

Supplementary Figure 1. Leave-one-out analysis results of *Bifidobacteriales(order)*, *Bifidobacteriaceae(family)*, and, *Bifidobacterium(genus)*.
